# Effects of prebiotic supplement on gut microbiota, drug bioavailability, and adverse effects in patients with colorectal cancer at different primary tumor locations receiving chemotherapy: study protocol for a randomized clinical trial

**DOI:** 10.1186/s13063-023-07137-y

**Published:** 2023-04-12

**Authors:** Ya Chen, Xiaowei Liao, Yanmin Li, Hong Cao, Feng Zhang, Bojian Fei, Chuanqing Bao, Huaxiang Cao, Yong Mao, Xiaoping Chen, Xiang Gao, Wei Zhao, Jianmin Xu

**Affiliations:** 1 Department of Endocrinology, Affiliated Hospital of Jiangnan University, Wuxi, 214122 China; 2 State Key Laboratory of Food Science and Technology, School of Food Science and Technology, Jiangnan University, Wuxi, 214122 China; 3 Department of Nutrition, Affiliated Hospital of Jiangnan University, Wuxi, 214122 China; 4 Department of Gastrointestinal Surgery, Affiliated Hospital of Jiangnan University, Wuxi, 214122 China; 5 Department of Oncology, Affiliated Hospital of Jiangnan University, Wuxi, 214122 China

**Keywords:** Colorectal cancer, Primary tumor locations, Gut microbiota, Adverse effects, Drug bioavailability, Prebiotic

## Abstract

**Background:**

The prevalence of colorectal cancer (CRC) worldwide is a huge challenge to human health. Primary tumor locations found to impact prognosis and response to therapy. The important role of gut microbiota in the progression and treatment of CRC has led to many attempts of alleviating chemotherapy-induced adverse effects using microecologics. However, the underlying mechanism of the difference in the prognosis of different primary tumor locations and the synergistic effect of prebiotics on chemotherapy need to be further elucidated. This study aims to explore the differences in tumor microbiota and examine the effectiveness of xylooligosaccharides (XOS) on gut microbiota, adverse effects, and bioavailability of chemotherapy drugs in CRC patients at different primary tumor locations.

**Methods:**

This is a double-blinded, randomized, parallel controlled clinical trial. Participants with left-sided CRC (LSCRC, *n* = 50) and right-sided CC (RSCC, *n* = 50) will randomly allocated to prebiotic group (*n* = 25) or control group (*n* = 25) and will receive either a daily XOS (3 g/day) or placebo, respectively, for 12 weeks. The primary outcomes will be the differences in the mucosa microbiota composition at different tumor locations and differences in gut microbiota composition, adverse effects, and blood concentration of capecitabine posttreatment. The secondary outcomes will include other blood indicators, short-chain fatty acids (SCFAs) concentration, quality of life, and mental health.

**Discussion:**

This study will reveal the potential benefits of prebiotic for improving the gut microbiota composition, alleviating the adverse effects, and improving the efficacy of chemotherapy in patients with CRC. In addition, this study will provide data on the different distribution of tumor microbiota and the different changes of gut microbiota during treatment in LSCRC and RSCC, which may provide novel insights into personalized cancer treatment strategies based on primary tumor locations and gut microbiota in the future.

**Trial registration:**

Chinese Clinical Trial Registry (www.chictr.org.cn): ChiCTR2100046237. Registered on 12 May 2021.

## Introduction

Colorectal cancer (CRC) is a common malignant tumor of the gastrointestinal tract that seriously affects the health of the digestive system. And the development of this tumor is a complex and multi-step process, from small dysplastic lesions of normal colorectal mucosa, through adenomatous polyps, to carcinoma in situ [1]. Based on location, CRC can be classified into left-sided CRC (LSCRC) and right-sided CC (RSCC). RSCC is defined as any tumor occurring in the cecum, ascending colon, hepatic flexure, or first two thirds of the transverse colon, while the LSCRC is defined as any tumor occurring in the latter one third of the transverse colon, splenic flexure, descending colon, sigmoid colon and rectum [2]. Worldwide, colorectal cancer (CRC) has become the third-ranked cancer in incidence and the second-ranked in mortality. It is estimated that 935,000 people died of CRC in 2020 according to the 2020 Global Cancer Statistics [3]. Lacking of physical activity, obesity, and diet were all independent factors related to the risk of CRC [4]. Some studies suggest that factors causing the imbalance of intestinal flora may also be significant to the carcinogenesis of CRC [5, 6]. Besides, different primary tumor locations differ in incidence, pathogenesis, clinical characteristics, survival prognosis, molecular biological characteristics, and gut microbiota [7]. Several studies have demonstrated that patients with LSCRC had a significantly better prognosis compared with those with RSCC [8–11].

Increasing evidence supports the character of intestinal flora in the pathogenesis and treatment of CRC. *Fusobacterium nucleatum* adheres, invades, and induces carcinogenic and inflammatory responses via its unique FadA adhesion to activate the growth of CRC cells [12]. Other than the direct effect of specific bacteria on partial tissues, the broader bacterial community may regulate the risk and progression of CRC via competitive rejection and other mechanisms. Gut microbiota-dependent short-chain fatty acids (SCFAs) generation fermented from dietary fiber exert protection against colorectal tumorigenesis in rodents [13].

At present, cytotoxic drugs are generally applied for postoperative adjuvant therapy or advanced CRC patients, usually accompanied by a series of adverse effects (such as constipation, diarrhea, nausea, and bone marrow suppression) and individual differences in drug efficacy [14, 15]. In recent years, with the rise of “pharmacomicrobiomics,” the significance of the intestinal flora in regulating the host response to chemotherapeutic drugs is increasingly recognized [16]. Intestinal flora can modulate the anti-tumor activity of chemotherapy drugs [17]. The killing effectiveness of 5-Fu on CRC can be enhanced by bacterial-derived urolithin A [18]. Importantly, proteobacteria can promote the deglycosylation of capecitabine [19]. Moreover, some studies have reported that probiotics/prebiotics can reduce the adverse effects of chemotherapy. *Lactobacillus casei* variety *rhamnosus* plays a preventive role in FOLFOX-related intestinal mucositis in CRC-bearing mice [20]. In CRC patients, *L. rhamnosus* GG consumption reduced the incidence of 5-FU-induced severe diarrhea and abdominal discomfort compared with guar gum fiber [21]. Lu et al. found that synbiotics (containing a variety of probiotics and prebiotic ingredients) can help relieve postoperative chemotherapy-related adverse effects, further improving the quality of life (QoL) in CRC patients [22]. Furthermore, accumulating evidence demonstrated that prebiotics, such as non-digestible oligosaccharides, can reverse chemotherapy-induced intestinal dysbiosis through selective colonization of the probiotic bacteria [23]. In Peng’s trials, compared to those who received enteral nutrition therapy alone, surgical patients supplied with prebiotics experienced shorter durations of hospital stay and had reduced levels of inflammatory cytokines [24]. Hence, targeting the gut microbiota in clinical practice to modulate the efficacy and toxicity of chemotherapeutic drugs has a promising future.

Although there have been some reports on the effects of microecologics on modulating intestinal flora and reducing adverse effects in patients with CRC during chemotherapy, they are still very limited. Through our current knowledge, using microecologics to interfere with the bioavailability of chemotherapeutic agents in a microbiota-dependent manner has not been reported, as well as differences in the intervention effect of prebiotics on the intestinal flora of CRC patients at different primary tumor locations.

Xylooligosaccharide (XOS) is a mixture of oligosaccharides consist of 2–9 xylose units connected by β-1,4-glycosidic bonds [25, 26], which can scape digestion by host enzymes in the small intestine and enter the large intestine directly, and served as major substrates for gut bacterial growth. XOS can be utilized by beneficial bacteria first in the intestine, enriching bifidobacteria and lactobacillus, and inhibiting the proliferation of harmful bacteria such as *E. coli and Salmonella enteritidis* to reduce the production of harmful substances [27, 28]. The proliferation effect of XOS on bifidobacteria is about 20 times that of other functional oligosaccharides, with higher selectivity [29]. XOS can facilitate the generation of SCFAs such as acetic acid, propionic acid, and butyric acid [30], as well as other organic acids such as lactic acid, succinate, formic acid, isobutyric acid, and isohexanoic acid [31], which play important roles in preventing various intestinal diseases. XOS was found to increase the moisture content of stool and improve constipation [32]. In addition, XOS have a strong ability to adsorb pathogens, thereby preventing diarrhea [33]. Evidence from in vitro and animal research indicates that XOS play roles in inhibiting the secretion of inflammatory cytokines and have an effect on anti-inflammatory and anti-tumor [34, 35]. Chronic toxicology studies on XOS show that it is safe and reliable with no toxicity in humans, dogs, rodents, and other animals [36–38].

## Objectives

Firstly, this study aims to explore the difference of tumor microbiota and the different gut microbiota changes during treatment between LSCRC and RSCC. Moreover, another goal of the trial is to investigate the effect of XOS on (i) increasing the bioavailability of cytotoxic drug, (ii) reducing the adverse effects of chemotherapy, and (iii) improving the QoL of CRC patients by modifying gut microbiota.

## Methods and analysis

### Study design

**Fig. 1:**
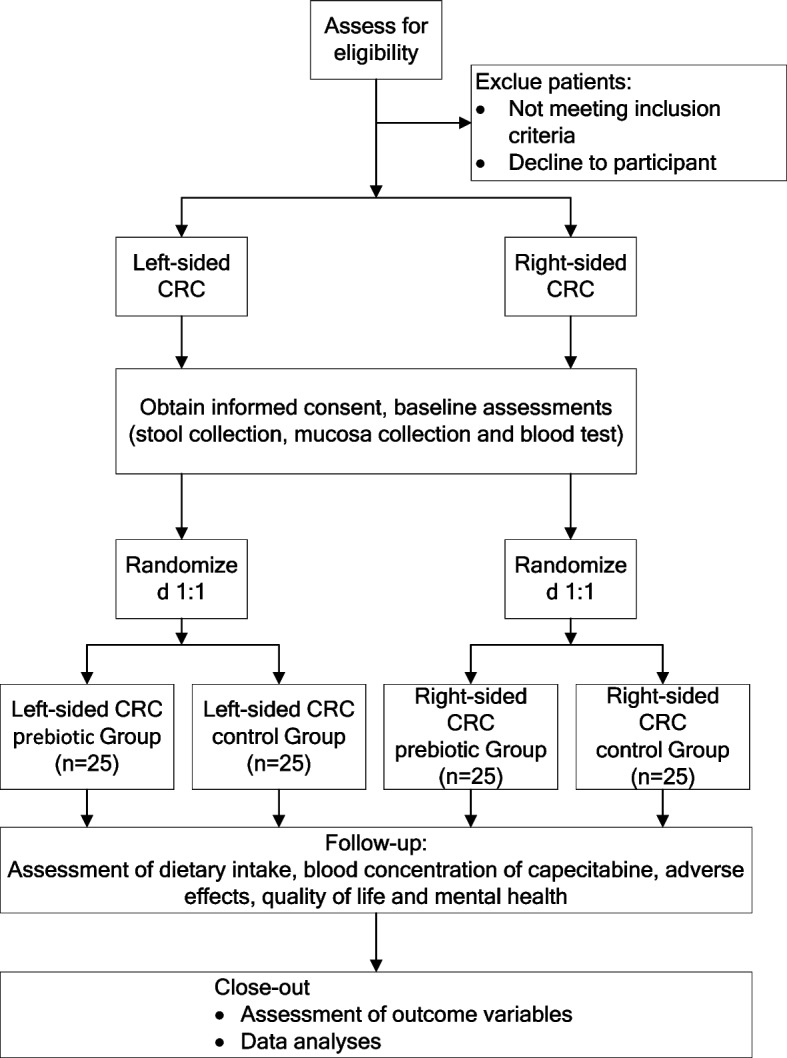
Study flowchart

This study is a single-center, randomized, parallel controlled clinical trial conducted at the Affiliated Hospital of Jiangnan University in Wuxi, China. Participants with LSCRC and RSCC will receive prebiotic or placebo intervention during postoperative chemotherapy for 3 months. The study flow is shown in Fig. 1. Study procedures and data collection are shown in Table 1 based on SPIRIT (Standard Protocol Items: Recommendations for Interventional Trials) [39].

**Table 1:**
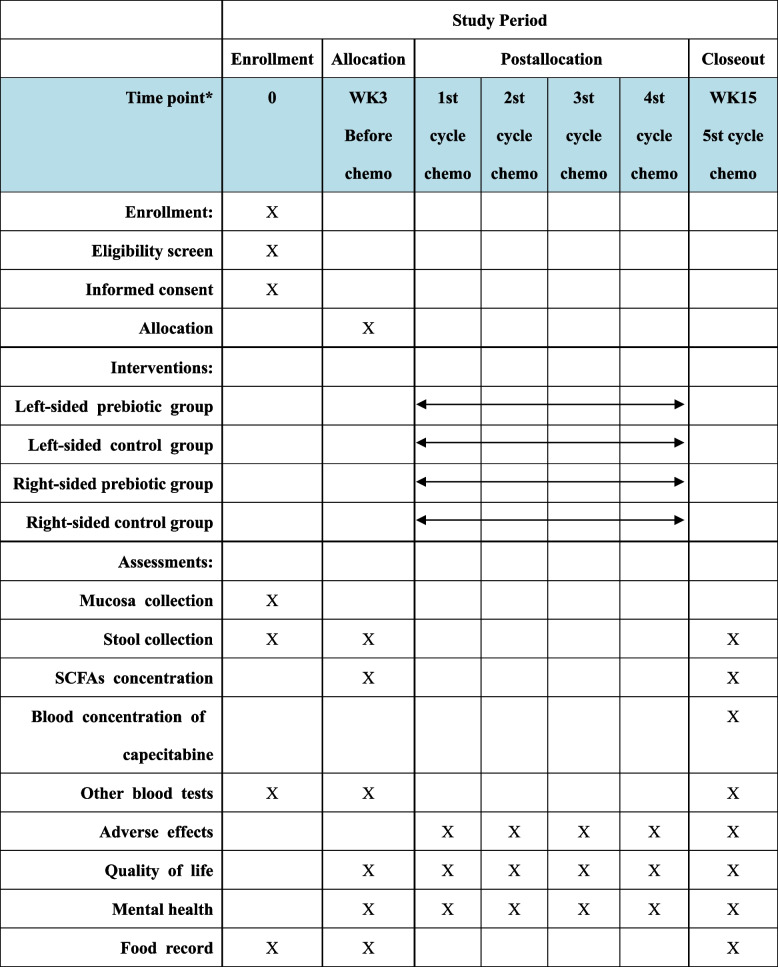
SPIRIT diagram: schedule of enrollment, interventions, and assessments

*The experiment takes 12 weeks, and every cycle of chemotherapy takes 3 weeks

### Eligibility criteria

#### Inclusion criteria

1. Patients diagnosed with primary CRC who will be treated with XELOX chemotherapy;
2. Patients aged 18–80 years;
3. Patients with an Eastern Cooperative Oncology Group (ECOG) performance status score of 0 or 1, absolute number of neutrophils ≥ 1.5 × 10^9^/L, platelets ≥ 100 × 10^9^/L, serum creatinine ≤ 1.5 times upper limit of normal (ULN), total bilirubin ≤ 1.5 times ULN, aspartate transaminase/alanine transaminase ≤ 2.5 times ULN and carcinoembryonic antigen within the normal range;
4. Patients have not received preoperative neoadjuvant radiotherapy and chemical treatment;
5. Written informed consent.

#### Exclusion criteria

1. Suffer from other tumors at the same time;
2. Patient has other digestive tract diseases except gastrointestinal tumors, such as inflammatory bowel disease, acute gastroenteritis, etc.;
3. Those who have taken antibiotics, drugs, or food containing probiotics within 6 months;
4. Those with active infections or those with mental illness, cardiovascular and cerebrovascular diseases, severe cardiopulmonary dysfunction, and other serious diseases that are not suitable for chemotherapy;
5. Those who have undergone multiple courses of chemotherapy, extensive radiotherapy, advanced age, bone marrow metastasis, severe infection, adrenal insufficiency, and severe illness;
6. Women during pregnancy;
7. Patients who cannot receive treatment on time and cannot cooperate fully.

#### Drop out criteria

1. Patients who violate medication regimen in the chemotherapy treatment;
2. Patients who receive antibiotics, probiotics or other prebiotics during the intervention;
3. Individuals who do not take the prebiotics consistently;
4. Individuals who suffer complications that may be caused by prebiotics;

### Selection and recruitment

Posters and leaflets will be accessible in the department of gastrointestinal surgery in the hospital so that interested candidates with CRC can get in touch with investigators to participate in the screening process. In addition, according to their patient charts, inpatients who meet inclusion criteria will be inquired about their intention for recruitment in the hospital. The eligible participants will be invited for the first visit, and two trained investigators will explain the study procedures in detail to them: (1) the purpose of this study, whether there are risks and discomforts, whether it needs to be paid, whether it is completely voluntary, etc.; (2) the types of samples collected in this study and the details of the collection; (3) the personal information of the subjects, research-related measurement indicators, and physical examination information will be kept confidential. Informed consent will be voluntarily signed by individuals who agree to participate in this study. The participants will visit the hospital for postoperative chemotherapy about 3 weeks after surgery. Since the chemotherapy regimen will change with the cancer stage, only participants who receive XELOX chemotherapy (oxaliplatin + capecitabine) will continue to participate in this trial.

### Information questionnaires and physical evaluation

The personal information of the subjects will be conducted through basic information questionnaires, including body mass index, age, gender, and dietary habit. And these data were obtained in the form of an interview. Meanwhile, physical examination (height, weight, blood pressure, heart rate, etc.) is performed before the allocation.

### Allocation

#### Sequence generation

Each patient, regardless of the study group allocated, will receive 3 g of prebiotic or placebo on the basis of standard adjuvant chemotherapy. Once a participant meets the enrollment conditions and signs the informed consent, they can open one envelope in the order of enrollment time. Prebiotic and placebo will be packaged in opaque bags without any graphics, and their appearance and smell are the same. A third party who has no direct involvement will stick the “A” and “B” codes on the packaging boxes.

#### Randomization and blinding

After signing the informed consent form and complete baseline questionnaires, patients with LSCRC (*n* = 50) and RSCC (*n* = 50) will be randomly divided into prebiotic group (*n* = 25) and control group (*n* = 25) in a 1:1 allocation ratio, respectively. The random number will be generated by a computer and sealed in envelopes by an investigator who will not be involved in running the study and placed in a safe place. Before data analysis, participants and investigators will not know the contents of the bags, allocation, and study treatments.

#### Allocation concealment

As it is a randomized controlled study, the patients are blinded. The unblinding occurs if a patient decides to leave the protocol prematurely.

#### Auditing

The audit process will be done independently from investigators and the sponsor.

#### Interventions

Participants in the prebiotic group (*n* = 25 per group) will be asked to consume prebiotic (XOS, 3 g/day) along with routine capecitabine therapy. Participants in the control group (*n* = 50 per group) will be asked to consume placebo (maltodextrin, 3 g/day). Prebiotics and placebo are white powder, packed in 3g/ bag. Since maltodextrin is colorless and tasteless after dissolution, it can reduce the chance of the placebo effect interfering with results. Each prebiotic bag (produced by Heagreen Company, Henan, China) contains 40.64% xylobiose, 27.75% xylotriose, 14.16% xylotetraose, 7.14% xylopentaose, and 7.8% xylohexaose. Each box contains 30 bags. Participants will be instructed to mix one bag prebiotic or placebo with water and consume it every day. Participants will be given a box of prebiotics each time they visit the hospital for chemotherapy (the supplementation will be delivered every 3 weeks). The intervention will last for 12 weeks. Participants will be monitored for prebiotic consumption at each delivery of prebiotics and weekly telephone follow-up. The adherence to treatment of participants will be checked by counting the number of the recycled package of prebiotics and placebo. Throughout the intervention, volunteers will be required to maintain their lifestyle, diet, as well as medications. They will be asked to collect stool and blood samples before (3 week) and after (15 week) the intervention and complete questionnaires in each chemotherapy cycle. The record of data will be conducted by a professional and the reliability of the data will be checked by another professional in time.

#### Sample size calculation

Since there is no clinical trial assessing the effect of intestinal flora on the bioavailability of chemotherapy drugs and the intervention endpoints of intestinal flora are currently unable to determine, the sample size is estimated based on the data from a previous clinical trial [22] which assessed the effects of synbiotics intervention on chemotherapy-related adverse effects (including diarrhea, appetite loss, nausea, and vomiting) for CRC patients received post-operative chemotherapy, showing that 20 volunteers per group had 90% power at an alpha level of 0.05 to detect significant differences. Combined with the above data from the clinical trial, the sample size in this study will be calculated statistically by the Power Analysis and Sample Size Software (PASS). Considering the long duration of the study (3 months) and the expected withdrawal of participants during the intervention, we plan to recruit *n* = 25 participants per group (*n* = 100 total).

#### Data collection and sample handling

The overall study design is depicted in Fig. 1. Assessment of participants will be conducted at the perioperative (baseline = week 0), pre-treatment (week 3), post-treatment (week 15), and in each chemotherapy cycle. The primary outcomes will be the differences in the microbiota composition at different CRC tumor sites and differences in gut microbiota composition, adverse reactions, and blood concentration of capecitabine in patients with CRC treated for 12 weeks with XOS or placebo. The secondary outcomes will include blood indicators (hepatic and renal function, blood glucose, blood lipid, and inflammatory cytokines), SCFAs concentration, QoL, and mental health.

#### Gut microbial composition

Tissue samples including tumor, para-carcinoma mucosa (2 cm away from the tumor), and normal mucosa (as far as possible from the tumor) will be obtained from LSCRC and RSCC participants during surgery. Each tissue will be cut into small pieces with a volume of 1 cubic centimeter and put into the cryopreserved tubes. After being frozen in liquid nitrogen, the tissue will be stored in the − 80 °C refrigerator for later use.

The collection of feces will be carried out 24 h before or after blood sample collection on the day of ending the treatment. A total of 6 g stool samples from each participant will be collected in the morning for intestinal flora examination at baseline prior to preoperative bowel preparation and at 3 weeks (pre-treatment) and 15 weeks (post-treatment). Briefly, participants will be instructed to (1) exhaust urine before placing disposable stool tray to prevent contamination of stool, (2) pass stool on the tray, and (3) use a sterile spoon to dig up about 2g stool from middle and posterior segment of stool and inserted into a sterilized screw cap containers marked with the participant’s code and sampling date. For the purpose of reducing the change of microbiota composition in the stools, samples will be temporarily stored in a foam box with ice packs and transferred to the – 80 °C refrigerator in the laboratory within 2 h. The collection of feces will be carried out at the residence, and the patients will bring it to the researcher in the hospital.

The total DNA of frozen samples will be extracted via QIAamp (QIAGEN) stool DNA kit according to manufacturer’s instructions. DNA integrity and size will be assessed by the NanoDrop ND-1000 spectrophotometer (Thermo, USA) and 1% agarose gel electrophoresis. DNA samples will be kept at – 80 °C for later use. The bacterial 16S rRNA V3–V4 regions will be amplified by polymerase chain reaction (PCR). High-throughput sequencing will be performed on an Illumina platform.

A sequence similarity threshold of 97% will be used to select operational taxonomic units (OTUs) and then perform taxonomy assignments via the Usearch platform (version 7.1 http://drive5.com/uparse/). Principal coordinates analysis (PCoA) on weighted UniFrac distances will be measured on all OTUs by QIIME. Alpha diversity will be calculated through the Shannon index, Simpson index, and Chao1 metrics using mothur (version v.1.30.1 http://www.mothur.org/wiki/Schloss_SOP#Alpha_diversity). Linear discriminant analysis (LDA) will be performed on the analysis software LEfSe (http://huttenhower.sph.harvard.edu/galaxy/). Subsequently, the communities or species that have significant differences in the sample division will be selected.

#### Stool SCFA profiling

Stool sample aliquots of 50 mg g of dry weight each will add with 50 μL phosphoric acid, 100 μL isohexanoic acid solution, and 400 μL diethyl ether to homogenize. After centrifugation, the supernatant will be separated for gas chromatography-mass spectrometry (GC-MS) analysis. Stool SCFA profiling will be conducted using an Agilent Technology 6890 GC with autosamplers and 5973 MS Detection and Chemstation Data System (Agilent, Singapore). Chromatographic conditions will be as follows: Agilent HP-INNOWAX capillary column (30 m × 0.25 mm ID × 0.25 μm); shunt injection, injection volume 1μL, shunt ratio 10:1; injection port temperature of 250 °C; ion source temperature of 230 °C; initial oven temperature of 90 °C, 10 °C/min up to 120 °C, 5 °C/min up to 150 °C, 25 °C/min up to 250 °C, and keep at 250 °C for 2 min. Helium will be used as gas carrier at a constant flow rate of 1 mL/min.

#### Blood tests

Venous blood samples will be collected before preoperative bowel preparation, at 3 weeks (pre-treatment) and 15 weeks (post-treatment). After fasting for 12 h, venous blood will be collected into a coagulating tube in the morning and centrifuged at 3000 rpm for 10 min for obtaining the upper serum (Thermo, USA). In addition to some of the usual blood biochemical indexes, we will use commercial enzyme-linked immunosorbent assay (ELISA) kits to assess the levels of inflammatory cytokines, including tumor necrosis factor-α (TNF-α), interleukin-1β (IL-1β), and interferon gamma (IFN-γ). Cellular immune indices, including T lymphocytes (CD3+), helper inducer T cells (CD3+CD4+), and suppressor/cytotoxic T cells (CD3+CD8+), will be measured by BD-FACS Canto II flow cytometer (Becton Dickinson, USA).

For the purpose of evaluating the bioavailability of chemotherapy drugs in participants, the blood concentration of capecitabine will be measured at 15 weeks (after intervention). On the third day of the fifth cycle of treatment (taking capecitabine for at least four meals), 5 mL venous blood samples will be collected 2 h after taking medicine in the morning, put into a tube with ethylene diamine tetraacetic acid (EDTA) anticoagulant, and sent to the pharmacy department of the hospital for testing immediately.

#### Adverse effects

Adverse effects, including nausea, vomiting, diarrhea, leukopenia, anemia, thrombocytopenia, diarrhea, oral mucosal reaction, peripheral neuropathy, hand-foot syndrome, pigmentation, and abnormal liver function, will be assessed in each chemotherapy cycle using the Common Terminology Criteria for Adverse Events (CTCAE) version 5.0 [40]. Patients will be informed of the types of all adverse effects prior to the start of the experiment. After the beginning of the experiment and before each chemotherapy treatment, all adverse effects will be questioned and recorded from grades I to IV according to severity in the office. There is no knowledge of the possibility of any discomfort or adverse event with this supplementation. Participants are asked to inform the investigators in time if there is any adverse event that may be associated with prebiotics. These adverse events will be recorded by investigators. Protocol modifications will be communicated to the ethics committee through an official modification request, and the participants will be notified by telephone.

#### Food guidance and records

The patients have the dietary pattern according to the nutrition guidelines for patients with colorectal cancer by the European Society for Clinical Nutrition and Metabolism (ESPEN) [41]. In order to minimize the interference of personal diet on the gut microbiota and record changes in dietary habits in time, volunteers will be asked to complete a 72-h food record each time a stool sample is collected. Before completing the food record, food record forms with filling instructions and food-weighing electronic scales will be distributed to patients in advance. The professionals will also teach them how to fill out forms. They need to describe all foods and beverages consumed in detail including ingredients, weight, and cooking style. Daily nutrient intakes will be calculated based on the China Food Composition 2009 [42].

#### Health-related QoL

QoL will be assessed in each chemotherapy cycle using Chinese version of QoL questionnaire-caner 30 (QLQ-C30) formulated by the European Organization for Research and Treatment of Cancer. The questionnaire has 30 items, including 5 functional scales, 3 symptom scales, 6 individual measures items, and a global health [43].

#### Mental health

The prevalence of anxiety and depression in CRC patients is 13–57% [44], and one of the important factors is the adverse effects of chemotherapy [45]. Hospital Anxiety and Depression Scale (HADS), an effective questionnaire with 14 items, will be used to assess depression and anxiety in each chemotherapy cycle. Each item is on a 4-point scale and final scores are proportional to the degree of anxiety and depression.

#### Data analysis

The visiting time of the participants is in accordance with the treatment course, so as to facilitate the retention of the participants. The measured data for each visit will be collected on paper and then recorded electronically on a secure, locked office computer. The paper version of the data will be locked in a bookcase. Only the investigators running in the study will have access to the final study dataset.

The obtained data will be analyzed by the SPSS software V26.0 (IBM, USA). Continuous data will be expressed as mean ± standard deviation (SD), and categorical data will be expressed as the number of cases (*n*) and percentage (%). Differences in parameter continuous variables and asymmetric variables between groups will be analyzed through independent samples *t* test and Mann-Whitney *U* test, respectively, using a paired *t* test to assess the effect of the intervention in each group. The normality of data will be assessed using the Kolmogorov-Smirnov test.

The results will be regarded as statistically significant if *p* values< 0.05.

## Discussion

CRC is one of the most prevalent cancers worldwide. Postoperative adjuvant chemotherapy for CRC is a helpful attempt in recent years. However, the adverse effects during chemotherapy and the inter-individual variation in drug efficacy are still relatively big problems. Moreover, the different primary tumor locations may result in differences in intestinal flora composition and prognosis. Efforts to find a kind of safe food with therapeutic effect and to evaluate its impact on patients with CRC at different primary tumor locations are critical. Studies have revealed the vital part of intestinal flora in the progression and treatment of CRC [5, 12, 46], and the concept of “pharmacomicrobiomics” is increasingly recognized [47]. Targeting intestinal flora, therefore, may modulate the efficacy and adverse effect of chemotherapy.

Both animal and human studies provide strong evidence that microecologics play essential roles in preventing chemotherapy-induced mucositis [48]. Bowen et.al. [49] have revealed the effects of VSL#3, a mixture of *Lactobacilli*,* bifidobacteria*, and* streptococcus*, on preventing diarrhea following chemotherapy with irinotecan in rats. Synbiotics have significantly reduced the occurrence of severe lymphopenia and diarrhea and increased levels of *Bifidobacterium* and *Lactobacillus* species and concentrations of SCFAs in esophageal cancer patients undergoing neoadjuvant chemotherapy [50]. Dietary supplementation of prebiotics (oligofructose and inulin) can enhance the efficacy of six cytotoxic drugs and prolong the lifespan of rodents with transplantable malignant tumors [51, 52]. XOS is a mixture of oligosaccharides which can be considered as a prebiotic [53]. XOS can modulate the diversity of intestinal flora, effectively multiply favorable bacteria such as bifidobacteria, and produce beneficial metabolites including SCFAs [54]. In addition, it has been reported that XOS are beneficial to type 2 diabetes mellitus [55], diarrhea [33], and constipation [32]. However, so far, there is no study evaluating the role of XOS consumption on the intestinal flora in patients with CRC.

This study will reveal the potential benefits of prebiotic for improving the gut microbiota composition, alleviating the adverse effects, and improving the efficacy of chemotherapy in patients with CRC. In addition, our study will provide data on the different distribution of tumor microbiota and the different changes of gut microbiota during treatment in LSCRC and RSCC, which may provide novel insights into personalized cancer treatment strategies based on primary tumor locations and gut microbiota in the future. 

## Trial status

This trial was registered at ClinicalTrials.gov, and this article is based on the 2nd version of the protocol published in September 2021. It was not submitted earlier to be published due to changes in the study status. The recruitments have begun beginning of June 2021, and due to the COVID-19 pandemic, the patient inclusions were delayed to resume by October 2022. The final inclusion will be terminated 1 March 2023.

## Protocol amendments

A second version of this protocol was conveyed in regard to the post-treatment visit (visit 2).

## Data Availability

Not applicable.

## Abbreviations

CRC: Colorectal cancer
SCFAs: Short-chain fatty acids
LSCRC: Left-sided colorectal cancer
RSCC: Right-sided colon cancer
QoL: Quality of life
XOS: Xylooligosaccharide
SPIRIT: Standard Protocol Items: Recommendations for Interventional Trials
ULN: Upper limit of normal
PCR: Polymerase chain reaction
OTUs: Operational taxonomic units
PCoA: Principal coordinate analysis
LDA: Linear discriminant analysis
GC-MS: Gas chromatography-mass spectrometry
ELISA: Enzyme-linked immunosorbent assay
TNF-α: Tumor necrosis factor-α
IL-1β: Interleukin-1β
IFN-γ: Interferon gamma
CD3+: T lymphocytes
CD3+CD4+: Helper inducer T cells
CD3+CD8+: Suppressor/cytotoxic T cells
EDTA: Ethylene diamine tetraacetic acid
CTCAE: Common Terminology Criteria for Adverse Events
QLQ-C30: QoL questionnaire-caner 30
HADS: Hospital Anxiety and Depression Scale

## Declarations

### Dissemination policy

A dissemination policy concerning the statistical data will be implemented to ensure that the official statistics are consistent, reliable, and comparable.
